# A mathematical model of human oesophageal motility function

**DOI:** 10.1098/rsos.250491

**Published:** 2025-08-20

**Authors:** Takashi Miura, Hiroshi Ishii, Yoshitaka Hata, Hisako Takigawa-Imamura, Kei Sugihara, Shin-Ichiro Ei, Xiaopeng Bai, Eikichi Ihara, Yoshihiro Ogawa

**Affiliations:** ^1^ Department of Anatomy and Cell Biology, Graduate School of Medical Sciences, Kyushu University, Fukuoka, Fukuoka Prefecture, Japan; ^2^ Hokkaido University Research Institute for Electronic Science, Sapporo, Hokkaido Prefecture, Japan; ^3^ Department of Medicine and Bioregulatory Science, Graduate School of Medical Sciences, Kyushu University, Fukuoka, Fukuoka Prefecture, Japan; ^4^ Department of Mathematics, Josai University, Sakado, Saitama Prefecture, Japan

**Keywords:** high-resolution manometry, mathematical modelling, oesophageal motility

## Abstract

Recent advances in various observation methods revealed several unique characteristics of oesophageal peristalsis and its disorders. However, a framework for understanding the oesophageal motility pattern is lacking. Here, we propose a simple mathematical model of the human oesophageal motility function. The model comprises central nervous system signals, enteric nervous system neurons (interneurons and motoneurons) and oesophageal smooth muscles. The neural function implements excitable dynamics at the oesophageal body and toggle-switch dynamics at the lower oesophageal sphincter. The local signal transmission in enteric nervous system and ‘the law of the intestine’ were also incorporated. The model behaviours can be understood using mathematical analysis, and we could reproduce the physiological dynamics of the normal oesophagus—deglutitive inhibition, unidirectional pulse transmission, restoration of lower oesophageal sphincter constriction and dilatation of the anal side of the pulse. In addition, we could reproduce various pathological motility patterns described in the Chicago classification by the combinations of parameter changes, which may provide insights into the possible pathogenesis of these disorders.

## 1. Introduction

### 1.1. Human oesophageal peristalsis

Peristalsis is a movement of the gastrointestinal tract that moves food forward [1]. Recent advances in observation technology, such as high-resolution manometry (HRM), reveal several unique characteristics of *oesophageal* peristalsis [2,3], which is different from well-studied intestinal peristalsis [4]. Oesophageal motility disorder causes non-cardiac chest pain and dysphagia, significantly reducing the patient’s quality of life and causing life-threatening disease conditions such as aspiration pneumonia [3].

### 1.2. Bistability of lower oesophageal sphincter region

**Figure 1.**
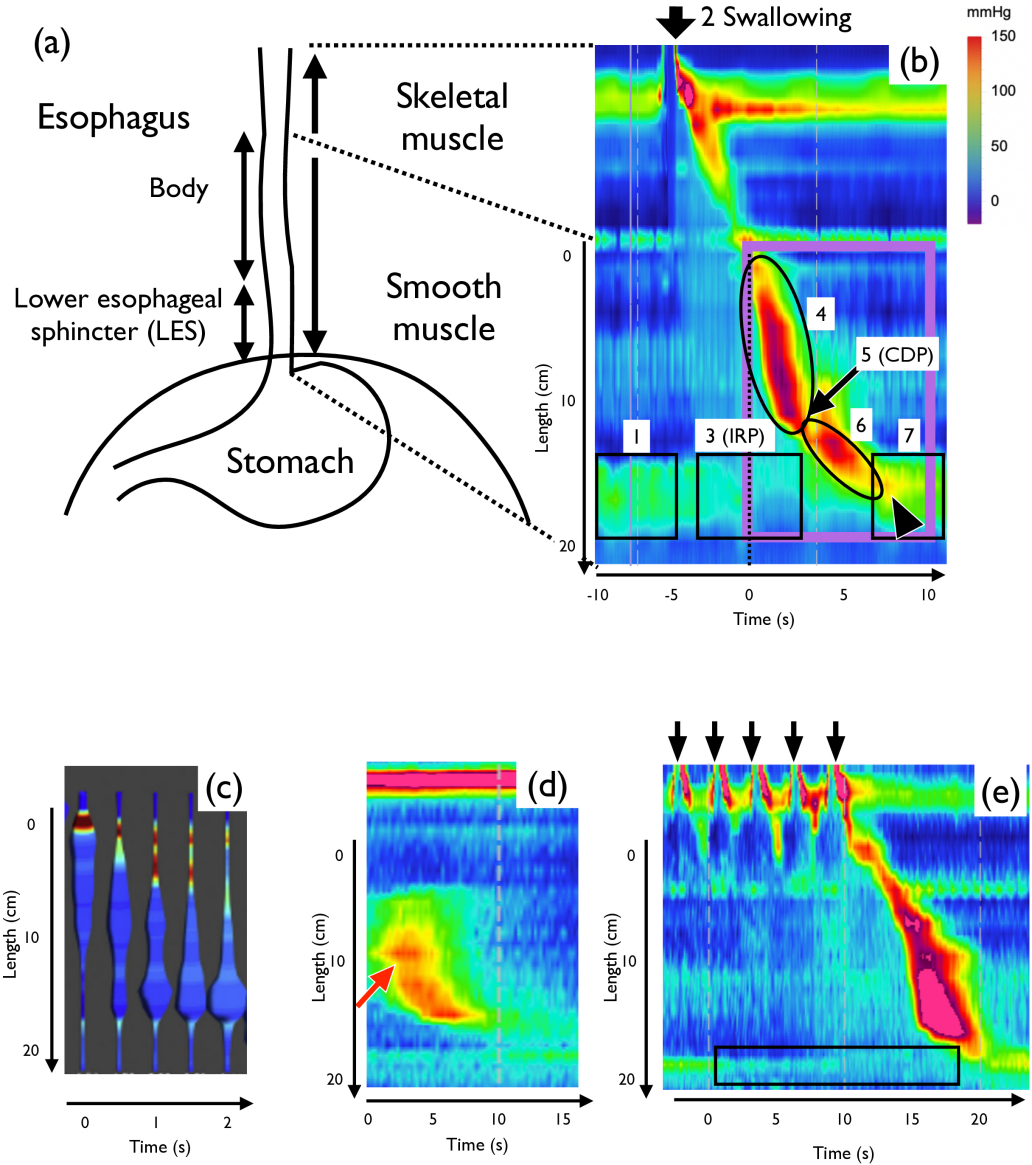
Human oesophageal peristalsis. (a) Schematic of the oesophagus and stomach, frontal view. (b) Typical pattern of oesophageal peristalsis in high-resolution manometry (HRM). The purple rectangle indicates the region of interest of this study. (c) A reconstructed cylindrical representation of impedance-derived distension and pressures (modified from [5]). (d) An example of secondary peristalsis that originates from the centre of the oesophagus (red arrow), showing unidirectionality of the pulse. (e) Multiple swallowing results in the transmission of only the last pulse. IRP remains normal (black rectangle). Pseudocolour represents oesophageal pressure.

One key characteristic of the oesophageal motility function is the lower oesophageal sphincter (LES, lowermost part of the oesophagus), which exhibits a toggle-switch-like (bistable) behaviour. In a resting state, LES is constricted (‘on’ state) to avoid gastric acid reflux (figure 1a,b(1), [6,7]). After swallowing (figure 1b(2)), the LES region is relaxed (‘off’ state) by the signal from the central nervous system (CNS), called deglutitive inhibition. As a result, the pressure inside the LES region is reduced (integrated relaxation pressure (IRP), figure 1b(3), [7]). The contraction pulse appears and moves at a constant speed (figure 1b(4)) up to a point called the contractile deceleration point (CDP) (figure 1b(5)), where the pulse speed is abruptly decelerated (figure 1b(6)). After the pulse passes through the LES, the LES is again constricted (‘on’ state) (figure 1b(7)).

Experimental observation shows that the LES region demonstrates a bistable behaviour: the LES is opened during belching to vent the air from the stomach. This transient LES relaxation is maintained for a long time until another pulse of constriction reaches LES [7]. This implies that the LES region exhibits two stable states, i.e. ‘on’ and ‘off’, which are changed by external signals.

### 1.3. Law of the intestine

During oesophageal peristalsis, the dilated region described in ‘the law of the intestine’ (excitation at any point of the gut excites contraction above, inhibition below) [8] appears in the distension–contraction plot, which detects oesophageal dilation using electronic estimation of the oesophageal diameter (figure 1c) [5,9–11].

### 1.4. Unidirectionality of the pulse

Another distinctive aspect is the unidirectionality of the pulse. Normal oesophageal peristalsis usually consists of a single moving pulse from the oral side to the anal side, and backward movement is rarely observed. When the peristalsis accidentally starts from the centre of the oesophagus (secondary peristalsis), only the forward moving pulse is observed (figure 1d) [12,13].

### 1.5. Repetitive swallowing

In addition, repetitive swallowing, also known as multiple rapid swallows (MRS), transmits only the last pulse (figure 1e) in the normal oesophagus. This is due to the deglutitive inhibition blocking the pulse generation by preceding swallowing. This MRS procedure is used as a test for normal oesophageal function.

### 1.6. Research summary

Although detailed descriptions of the dynamics of normal and abnormal oesophageal motility are accumulating, the theoretical framework that can reproduce these essential characteristics is lacking. One reason is that many of the characteristics are human-specific, and direct experimental manipulation to obtain physiological data is very difficult.

In the present study, we constructed a mathematical model that reproduces the normal and abnormal oesophageal motility patterns. At first, we focused on the lower two-thirds of the human oesophagus (purple rectangle in figure 1b) and formulated a mathematical model that reproduces the generation of unidirectional pulses, LES dynamics and ‘the law of the intestine’. We can systematically change the parameters to reproduce various oesophageal abnormalities described in the Chicago classification, indicating that these results may provide insights into the possible pathogenesis of these disorders.

## 2. Methods

### 2.1. The model

#### 2.1.1. Overview

**Figure 2.**
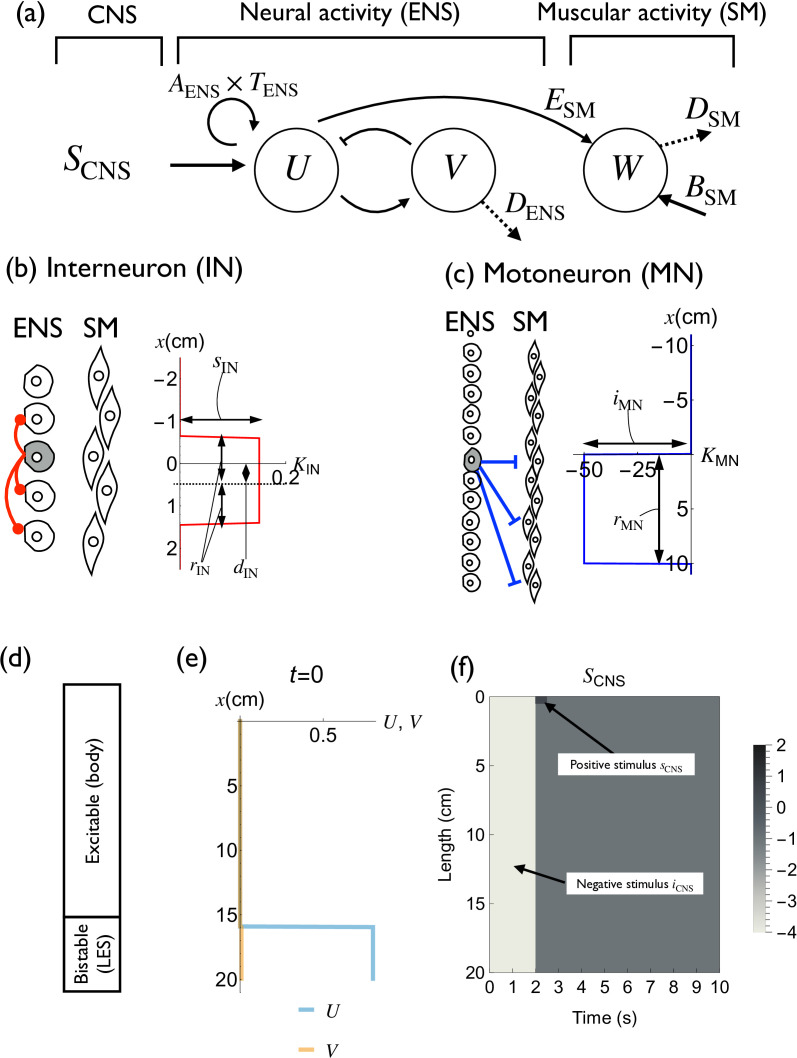
Model description. (a) The model consists of CNS signal ($S_{\text{CNS}}$), neural activity (U, V) and muscular activity (W). CNS, central nervous system; ENS, enteric nervous system; SM, smooth muscle. (b) Definition of the interneuron kernel $K_{\text{IN}}$. The kernel has a short-range ‘stimulatory’ region that deviates to the anal side. (c) Definition of the motoneuron kernel $K_{\text{MN}}$. The kernel consists of a long-range ‘inhibitory’ region, which implements the distension at the anal side (figure 1d). (d) Schematic of the difference between LES and oesophageal body. The oesophageal body is excitable, and the LES region is bistable. (e) Initial condition of U and V. (f) Definitions of positive and negative stimuli by single swallowing ($S_{\text{CNS}}$).

We used a model consisting of the CNS signal, the enteric nervous system (ENS) activity and the smooth muscle (SM) activity (figure 2a; §2.1.6). We used the modified FitzHugh–Nagumo equation, a widely used model of various electrophysiological phenomena [14], to model ENS activity. Since our model deals with the general status of neural activity and not an action potential or specific current, we modified the original FitzHugh–Nagumo equation to adjust to the observed behaviours.

(1) We shifted the equilibrium point to (0,0) by parameter substitution for analytical simplicity.
(2) We modified the V dynamics to avoid post-inhibitory rebound spike (PIRS, electronic supplementary material, appendix S6).
(3) Instead of using the diffusion term, we used the asymmetric convolution kernel to implement the local interaction within the interneurons of the ENS. It is experimentally observed that projections of neurons in ENS are asymmetric [15], so we introduced $d_{\text{IN}}$ to reflect this observation.

We also implemented the SM dynamics parameter W, which follows the ENS activity U (figure 2a) and receives descending inhibitory branches from motoneurons in the ENS [16].

#### 2.1.2. Central nervous system signal

Stimulation following the swallowing is implemented by $S_{\text{CNS}}(x,t)$ (equation (2.1), figure 2f). This parameter consists of positive stimulus $s_{\text{CNS}}$ at the oral edge and negative stimulus $i_{\text{CNS}}$ at the whole oesophageal body (deglutitive inhibition). $s_{\text{CNS}}$ is a locally induced stimulus by the passage of food at the beginning of the oesophageal body, and a negative stimulus $i_{\text{CNS}}$ is the stimulus to open LES and to inhibit the preceding pulse in MRS.

#### 2.1.3. Enteric nervous system activity

**Figure 3.**
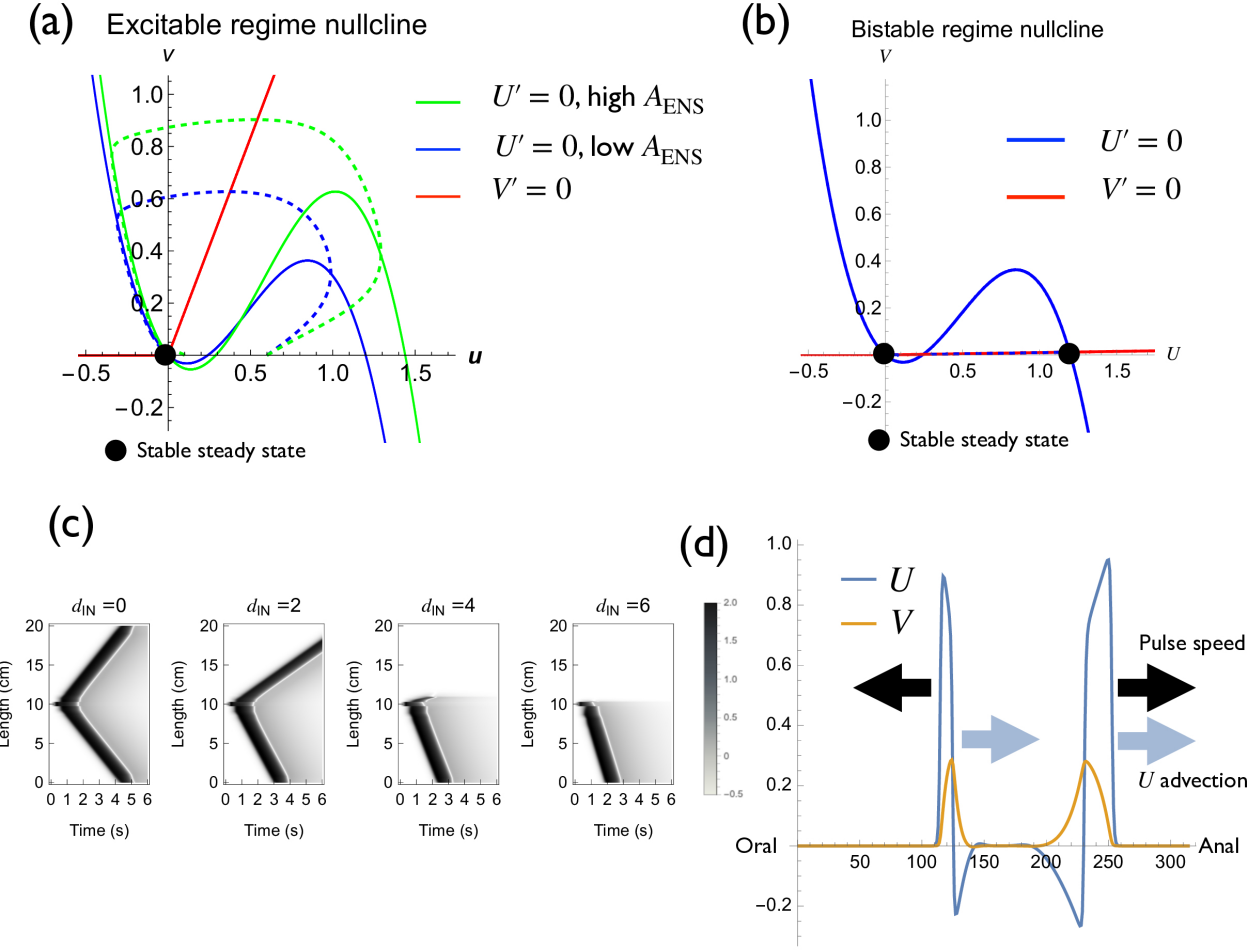
Dynamics of the model behaviour. (a,b) Implementation of excitability and bistability. (a) Nullclines (solid line) and temporal responses (dashed line) at the excitable region. Blue lines represent normal cases, and green lines represent pathologically high amplitude cases. (b) Nullcline (solid line) and temporal response (dashed line) at the bistable region. (c,d) Unidirectional pulse propagation. (c) Effect of d on unidirectional pulse propagation. (d) Intuitive explanation of the disappearance of a backward pulse.

*Implementation of excitability and bistability.* The ENS part is the modified FitzHugh–Nagumo model, designed to exhibit both excitable and toggle-switch (bistable) behaviours with the same model (U and V; figure 2d; electronic supplementary material, figure S2a). In the body region, stimuli below the threshold resulted in no response, whereas stimulation above the threshold induced a pulse generation (electronic supplementary material, material S3a). In the LES region, the ‘on’ state and ‘off’ state are changed if positive or negative stimuli exceed a certain threshold (figure 3b).

We defined neural dynamics as follows:


∂U∂t=−10(U(U−TENS(x)AENS(x))(U−2AENS(x))−0.5V)(2.1)+KIN(x)∗U+SCNS(x,t),(2.2)∂V∂t=r(U)−DENS(x)V.

U(x,t) and V(x,t) represent the neural activity of ENS at time t and location x. $T_{\text{ENS}}$ represents the threshold of the excitation (figure 2a). In some pathological states, $T_{\text{ENS}}$ at the LES region may have separate values. $A_{\text{ENS}}(x)$ is the parameter that controls the excitation amplitude (figure 2a). $D_{\text{ENS}}(x)$ represents the difference of the ENS dynamics between the body and LES of the oesophagus (figure 2d). We used $D_{\text{ENS}}(x)=100$ and $D_{\text{ENS}}(x)=0.6$ for LES and body regions, respectively (electronic supplementary material, figure S2a). As a result, LES region shows bistable behaviour while the body region is within the excitable regime (figure 2d). $S_{\text{CNS}}(x,t)$ represents the signal from CNS (figure 2f).


(2.3) $$\begin{array}{lrr} & r(U)=\text{max}(U,0) & \end{array}$$

is the rectifier function to implement the fact that hyperpolarization by negative stimulus $i_{\text{CNS}}$ does not induce a PIRS (electronic supplementary material, appendix S6).

*Spatial signal transmission.* Spatial signal transmission within the neural part is implemented by the interneuron kernel $K_{\text{IN}}(x)$, which is deviated to the anal side (figure 2b). Signal transmission from the ENS to the SM is implemented by the motoneuron kernel $K_{\text{MN}}(x)$ (figure 2c). We defined the kernel functions as follows (figure 2b,c):


(2.4)KIN(x)={0x<−rIN+dINsIN−rIN+dIN≤x≤rIN+dIN0rIN+dIN<x,(2.5)KMN(x)={0x<0iMN0≤x≤rMN0rMN<x.

∗ represents the convolution


(2.6) $$\begin{array}{lrr} & (K*U)(x,t)=\int K(x-y)U(y,t)dy. & \end{array}$$

The shape of $K_{\text{IN}}(x)$ reflects the experimentally observed spatial deviation of the interneuron connections [15]. The kernel consists of the asymmetric excitatory zone with amplitude $s_{\text{IN}}$, radius $r_{\text{IN}}$ and deviation $d_{\text{IN}}$, which generates the unidirectional travelling pulse (figure 2b). The shape of the kernel $K_{\text{MN}}(x)$ represents the effect of motoneurons that implement ‘the law of the intestine’. We set the inhibitory zone with amplitude $i_{\text{MN}}$ and length $r_{\text{MN}}$ (figure 2c) as experimentally observed [16].

#### 2.1.4. Smooth muscle activity

**Figure 4.**
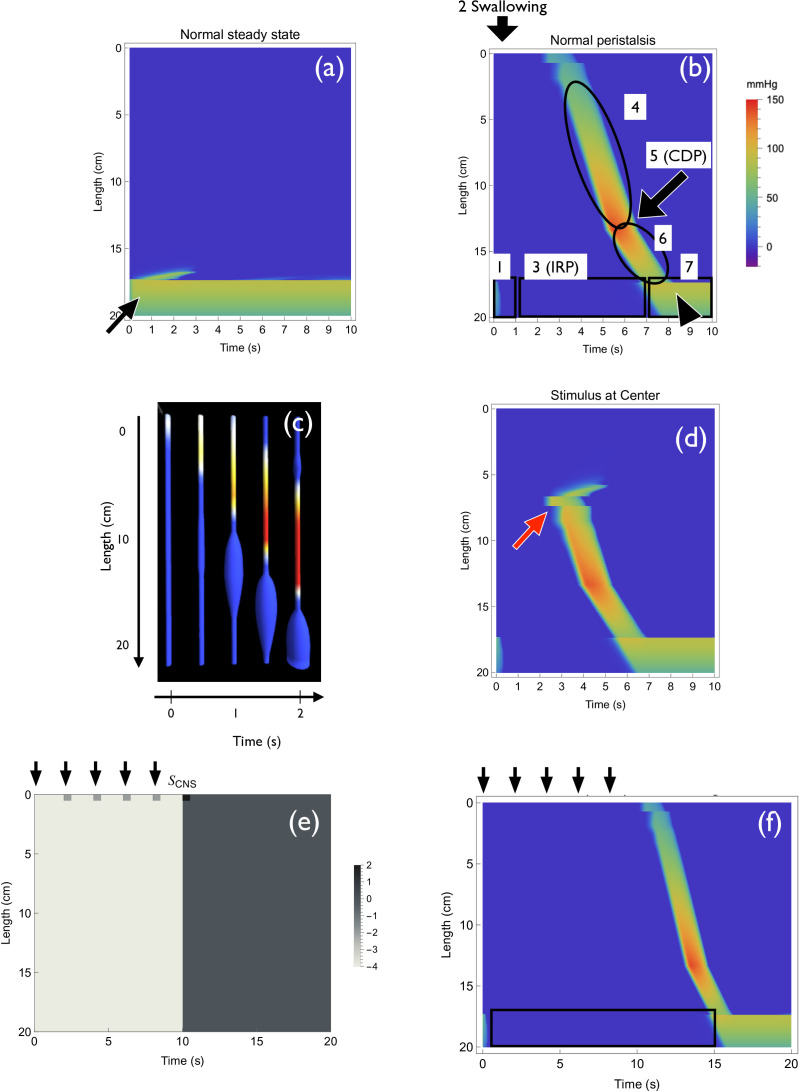
Numerical simulations of the normal oesophagus. (a) Numerical simulation results show a steady state of the oesophagus without stimulus. LES contraction is maintained. (b) Dynamics of a pulse after swallowing. The pattern reproduces the actual HRM pattern (figure 1b). (c) Distension–contraction plot view of the numerical simulation result. (d) Unidirectional transport of the pulse in the model. When the centre of the oesophageal body is stimulated, only a forward pulse is transmitted. (e) Definition of $S_{\text{CNS}}$ for repeated stimulus. Inhibitory signals from the next swallow mask the previous positive stimulus. (f) Result of multiple swallowing in the model. Only the last signal is transmitted. IRP remains normal (black rectangle).

Next, we defined muscular dynamics as follows:


(2.7) $$\begin{array}{rl} & \frac{\partial W}{\partial t}=(E_{\text{SM}}(x)U-D_{\text{SM}}(x)W+B_{\text{SM}}(x))+K_{\text{MN}}(x)*U+W_{\text{PEP}}(x,t), \end{array}$$

where W(x,t) represents the muscle contraction induced by the neural activity (figure 2a). $E_{\text{SM}}(x)$ represents the efficacy of neuromuscular signal transmission, $D_{\text{SM}}(x)$ is the decay constant of muscular excitation and $B_{\text{SM}}(x)$ is the basal smooth muscle excitation (electronic supplementary material, figure S2f). The final muscle contraction strength and duration can be modulated by changing $D_{\text{SM}}(x)$ or $E_{\text{SM}}(x)$. We used this characteristic to implement the gradual change in contraction strength before the CDP and subsequent decrease without changing the pulse speed (figure 4c; electronic supplementary material, figure S2). $W_{\text{PEP}}(x,t)$ is a function used to implement PEP. This function is normally zero but has a positive value when PEP exists (electronic supplementary material, figure S3h).

In addition, the effect of descending inhibitory branches of the motoneuron $K_{\text{MN}}(x)$ is defined to generate the dilatation region at the anal side of the U pulse.

The meanings of the model parameters are summarized in table 1. The parameter values were determined by the following procedure:

(1) We first established a model to reproduce the qualitative dynamics of oesophageal peristalsis using prior knowledge of FitzHugh–Nagumo model behaviour.
(2) Next, we numerically and mathematically analysed the model behaviours.
(3) The parameter values are optimized to reproduce the actual behaviour.

**Table 1.** Biological meanings of model parameters. Capital letters indicate values with spatial or temporal distribution. Lowercase letters indicate scalar values. The subscript describes the biological component of the parameter. The letter is defined as the first character of its function. For example, the stimulatory signal is indicated by s, and the inhibitory signal is denoted by i.

parameter	biological interpretation	reference
$D_{\text{ENS}}(x)$	distinction between oesophageal body and LES	[7]
$T_{\text{ENS}}(x)$	threshold of ENS neuron excitation	[17]
$A_{\text{ENS}}(x)$	amplitude of ENS neuron excitation	[18]
$E_{\text{SM}}(x)$	efficacy of neuromuscular signal transmission	[19]
$D_{\text{SM}}(x)$	decay constant of muscular excitation	[20]
$B_{\text{SM}}(x)$	basal smooth muscle excitation	[20]
$K_{\text{IN}}(x)$	interneuron projections in ENS	[15]
$r_{\text{IN}}$	range of interneuron projections in ENS	[15]
$s_{\text{IN}}$	strength of interneuron connectivity in ENS	[15]
$d_{\text{IN}}$	deviation of interneuron projections to anal size	[15]
$K_{\text{MN}}(x)$	motoneuron projections from ENS to smooth muscle	[16]
$r_{\text{MN}}$	range of inhibitory projections from ENS to smooth muscle	[16]
$i_{\text{MN}}$	strength of inhibitory projections from ENS to smooth muscle	[16]
$S_{\text{CNS}}(x,t)$	CNS signal induced by swallowing	[4]
$s_{\text{CNS}}$	amplitude of positive stimulus by food intake	[4]
$i_{\text{CNS}}$	amplitude of negative stimulus from CNS	[4]

Because direct measurements of variables U, V (neural activity) and W (smooth muscle activity) are not available experimentally, we adjust only their temporal dynamics. The details of the visualization methods are described in §2.1.6.

#### 2.1.5. Numerical implementation

The model was implemented by *Mathematica* Language (Wolfram Research Inc.) using the explicit Euler scheme. We used Δx=0.1 and Δt=0.01 for numerical simulations. We confirmed that changing Δx and Δt does not change the results. The relevant codes for this research work are stored in GitHub: https://github.com/miuraTakashi/EsophagealPeristalsisModel and have been archived within the Zenodo repository: https://doi.org/10.5281/zenodo.15854411.

#### 2.1.6. Visualization of model behaviour by high-resolution manometry and distension plot

*High-resolution manometry*. HRM does not directly represent all the characteristics of W(x,t). We converted the intraluminal pressure pattern W(x,t) into the HRM pattern as follows:

– Negative pressure values are standardized to zero because the distension region (figure 1c) cannot be detected in HRM (figure 1b).
– The x coordinate after CDP is rescaled to reproduce the observed slowdown of the pulse speed (figure 1b(6)). The reason why we choose this implementation is described in electronic supplementary material, appendix S2.

The visualization function of HRM is defined as follows:


(2.8) $$\begin{array}{r} HRM(x)=\left\{ \begin{array}{ll} r(W(x)) & x<x_{\text{CDP}}\\ r(W(x_{\text{CDP}}+0.5(x-x_{\text{CDP}})) & x_{\text{CDP}}\leq x, \end{array}\right. \end{array}$$

where r(W)=max(W,0) is the rectifier function defined in (2.3), which represents the characteristic of HRM that only the positive pressure is detected.

*Distension plot*. The distension plot represents muscle dilatation (W(x,t)<0) as the increased radius of a tube, and muscle contraction (W(x,t)>0) as the change in colour (figure 1c). We converted the intraluminal pressure pattern W(x,t) into the distension plot pattern as follows:

– The muscle dilation region (W(x,t)<0) is represented as the increase in radius of the thin tube. The radius of the contraction region (W(x,t)>0) is standardized to ($r_{\text{base}}$) because the contraction region is not observed in the distension plot (figure 4c).
– The muscle contraction region (W(x,t)>0) is visualized by the pseudocolour (figure 4c).

The radius $R_{\text{distention}}(x)$ and colour $C_{\text{distention}}(x)$ are defined as follows:


(2.9)Rdistention(x)=r(−W(x))+rbase,(2.10)Cdistention(x)=HRM(x).

HRM(x) and distension plot were plotted by colour, while U, V and W were plotted by greyscale.

### 2.2. High-resolution manometry

The human oesophageal motility patterns were assessed by HRM using a ManoScan Z (Given Imaging, Los Angeles, CA, USA). HRM was performed using a standardized protocol as previously reported [21,22]. In brief, after the basal condition without swallowing was recorded for 30 s, the patients were instructed to swallow as infrequently as possible and to breathe quietly and regularly. Next, the patients were asked to perform 10 swallows of 5 ml water at 1 min intervals in the supine position. The diagnosis was made using the 10 wet swallows based on Chicago Classification v. 3.0 [23]. For the MRS test, patients were instructed to swallow five times in rapid succession, typically at 2 s intervals.

**Figure 5.**
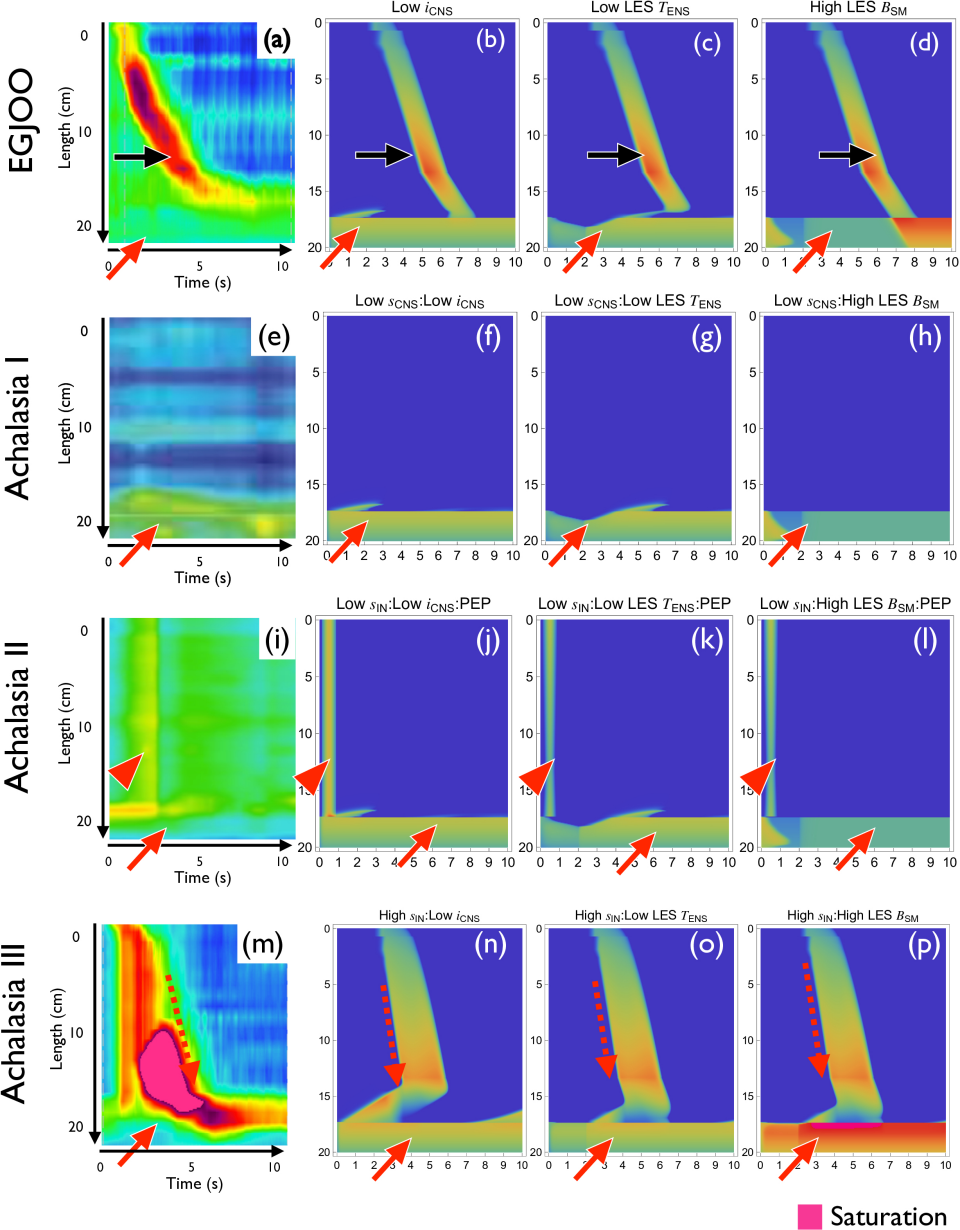
Reproduction of disorders of oesophagogastric junction (EGJ) outflow. (a–d) HRM pattern (a) and numerical simulations (b–d) of EGJ outflow obstruction (EGJOO). Normal pulse transmission (black arrows) and loss of LES relaxation (red arrows) are observed. (b) Low $i_{\text{CNS}}$. (c) Low $T_{\text{ENS}}$ at LES. (d) High $B_{\text{SM}}$ at LES. (e–h) HRM pattern (e) and numerical simulations (f–h) of type I achalasia. Lack of pulse transmission and loss of LES relaxation are observed. (f) Low $s_{\text{CNS}}$ and Low $i_{\text{CNS}}$. (g) Low $s_{\text{CNS}}$ and low $T_{\text{ENS}}$ at LES. (h) Low $s_{\text{CNS}}$ and high $B_{\text{SM}}$ at LES. (i–l) HRM pattern (i) and numerical simulation (j–l) of type II achalasia. Lack of pulse transmission, loss of LES relaxation and PEP are observed. Reduced LES relaxation (red arrows) and PEP are observed (red arrowheads). (j) Low $s_{\text{CNS}}$, low $i_{\text{CNS}}$ and PEP. (k) Low $s_{\text{CNS}}$, low $T_{\text{ENS}}$ at LES and PEP. (l) Low $s_{\text{CNS}}$, high $B_{\text{SM}}$ at LES and PEP. (m–p) HRM pattern (m) and numerical simulation (n–p) of type III achalasia. Reduced LES relaxation (red solid arrows) and premature contraction (red dashed arrowheads) are observed. (n) High $r_{\text{IN}}$ and low $i_{\text{CNS}}$. (o) High $r_{\text{IN}}$ and low $T_{\text{ENS}}$ at LES. (p) High $r_{\text{IN}}$ and high $B_{\text{SM}}$ at LES.

**Figure 6.**
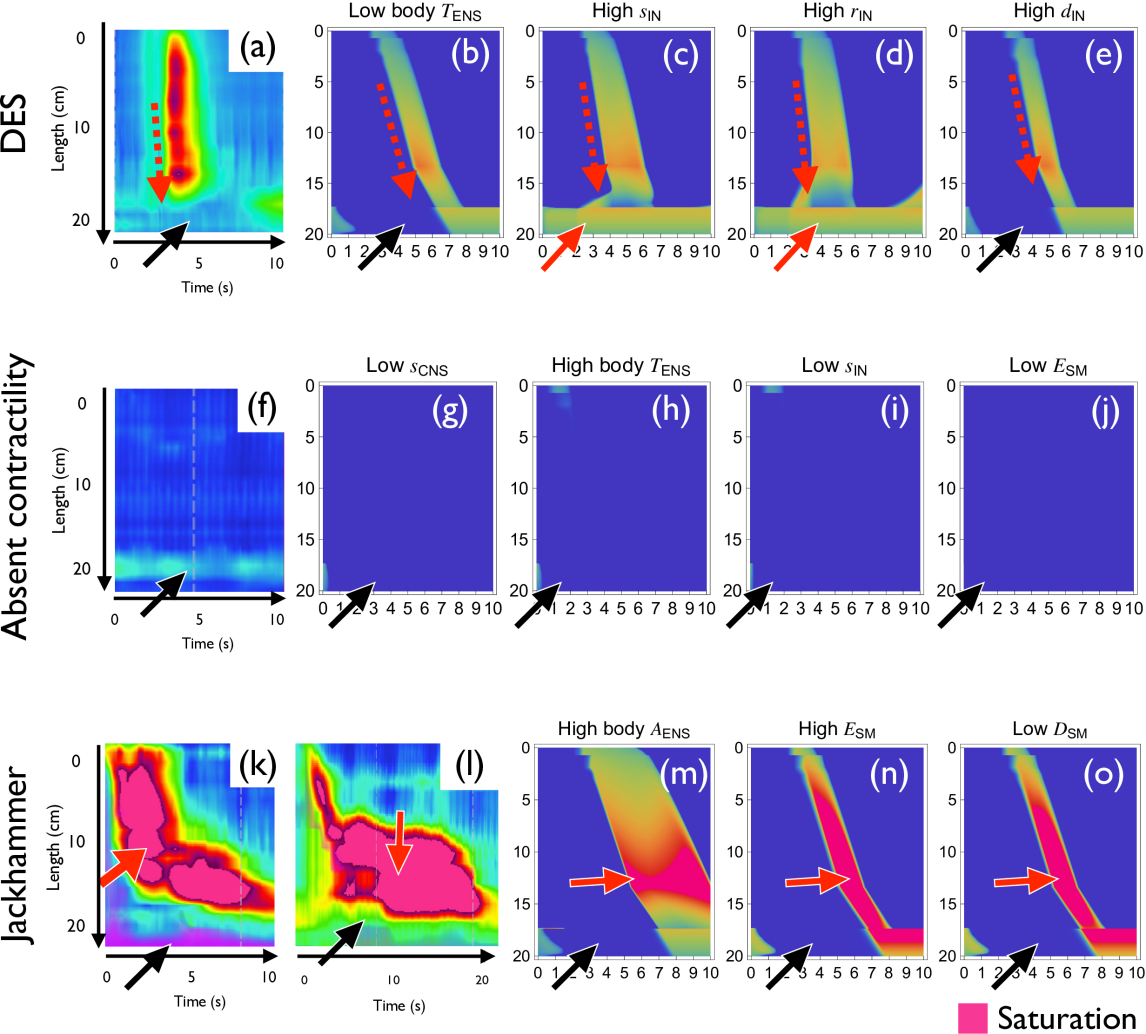
Reproduction of oesophageal body anomaly. (a–e) Distal oesophageal spasm. Normal LES relaxation (black arrows) and accelerated excitation wave (dashed red arrows) are observed. (a) HRM pattern [24]. (b) Low $T_{\text{ENS}}$ at body. (c) High $s_{\text{IN}}$. (d) High $r_{\text{IN}}$. (e) High $d_{\text{IN}}.$ (f–j) Absent contractility. The contraction pulse disappears. (f) HRM pattern. (g) Low $s_{\text{CNS}}.$ (h) High $T_{\text{ENS}}$ at body. (i) Low $s_{\text{IN}}.$ (j) Low $E_{\text{SM}}.$ (k–o) Jackhammer oesophagus. Strong contraction is observed. (k) HRM pattern of short-duration jackhammer oesophagus. (l) HRM pattern of long-duration jackhammer oesophagus. (m) High $A_{\text{ENS}}$ at body. (n) High $E_{\text{SM}}$. (o) Low $D_{\text{SM}}$.

Patients of typical disease types were selected as a representative case. A total of 11 samples were used in this study (figures 1b,d,e, 5a,e,i,m and 6a,f,k,l).

## 3. Results

### 3.1. Reproduction of normal oesophageal motility pattern

#### 3.1.1. Steady state

At first, we reproduced the steady state before swallowing—the LES region contracted while the other part of the oesophagus relaxed. Numerical simulation showed that the contraction of the LES region is maintained when peristalsis is absent (figure 4a, black arrow). This is because the relaxed state is stable in the body region, and both the relaxed and contracted states are stable in the LES region (§3.2.1).

#### 3.1.2. Pulse transmission

Next, we reproduced the transmission of the contraction pulse in normal peristalsis. We modelled the CNS signal by $S_{\text{CNS}}$ (figure 2f), which consists of negative ($i_{\text{CNS}}$) and positive ($s_{\text{CNS}}$) signals. The negative signal to the LES region relieves contraction (figure 1b(3)), and the positive signal to the oral edge of the oesophagus starts the pulse of peristalsis (figure 1b(4)). Numerical simulation showed that the pulse was transmitted from the oral to the anal direction (figure 4b(4)), changed speed at CDP (figure 4b(5); electronic supplementary material, appendix S2), and finally allowed the LES region to contract again (figure 4b(7)). A slight overshoot was observed in the LES region (figure 4b, arrowhead), which was also observed in HRM (figure 1b, arrowhead). Together, the simulation patterns faithfully reproduced the observed dynamics.

#### 3.1.3. Law of the intestine

It is known that the law of the intestine may also hold in the oesophagus (figure 1c). We observed the dilatation at the anal side of the contraction W(x,t) (figure 4c, electronic supplementary material, appendix S2.1.6), which reproduces the behaviour observed *in vivo* (figure 1c).

#### 3.1.4. Unidirectionality of the pulse transmission

To confirm whether our model can reproduce unidirectional transmission (figure 1d), we stimulated the centre of the oesophagus in the model (figure 4d, red arrow). The contraction pulse only moved in the anal direction (figure 4d).

#### 3.1.5. Multiple rapid swallows

Next, we reproduced the behaviour of MRS in the model. We assume that the signal from the CNS in the oesophagus is the sum of $S_{\text{CNS}}$ at multiple starting points (figure 4e). As a result, we could reproduce the dynamics of MRS in which only the last pulse is transmitted (figure 4f). This is due to the deglutitive inhibition of the positive signal.

### 3.2. Analyses of model behaviours

Next, we undertook numerical and mathematical analyses to understand the model behaviours.

#### 3.2.1. Bistability and excitability of ENS

We undertook phase plane analysis to understand excitable and bistable dynamics at the body and LES regions (figure 3a). In the excitable regime, the nullclines U and V (solid lines of blue and red) cross at the origin, the only stable steady state. If the stimulus to the U direction exceeds the threshold, the system is excited (figure 3a, dotted lines). The amplitude of U excitation corresponds to $2A_{\text{ENS}}(x)$. We can modulate the neural excitation amplitude by modifying $A_{\text{ENS}}(x)$ (figure 3a, green line). In the bistable regime, the phase plane analysis showed that the bistable regime has three steady states, one unstable and two stable (figure 3b). Numerical simulation of U and V dynamics at a single point confirmed the result. There are two stable steady states in the LES region, and the final steady state depends on the initial value (figure 3b).

#### 3.2.2. Generation of propagating pulse

We also undertook analyses of pulse transmission speed. At first, we undertook numerical simulations to observe the effects of interneuron parameters $s_{\text{IN}}$, $r_{\text{IN}}$ and $d_{\text{IN}}$ on the pulse speed (electronic supplementary material, figure S5a–c). In addition, we analytically derived the pulse speed using the reaction-diffusion approximation (electronic supplementary material, appendix S3). The numerical results of pulse speed were accurately predicted from mathematical analysis (electronic supplementary material, figure S5a–c).

#### 3.2.3. Unidirectional pulse transmission

We first numerically observed the relationship between the unidirectionality of the pulse and $d_{\text{IN}}$ (figure 3c). When $d_{\text{IN}}=0$, the pulse is bidirectional. By increasing $d_{\text{IN}}$, the counter-propagating pulse speed decreases, and the width of the pulse becomes thinner. When the $d_{\text{IN}}$ is larger than 2, the counter-propagating pulse disappears (figure 3c). According to this analysis, we chose parameter $d_{\text{IN}}$ above a certain threshold. In addition, by decreasing $d_{\text{IN}}$ and introducing stochasticity in $T_{\text{ENS}}$, we can reproduce a pathological condition in which a backward pulse is stochastically observed (electronic supplementary material, appendix S5, figure S6).

We then mathematically showed that we could not construct a pulse solution if the deviation exceeds a threshold defined by the model parameter $d_{\text{IN}}$ (electronic supplementary material, appendix S5).

The mechanism of unidirectional transmission can be understood intuitively. $d_{\text{IN}}$, the deviation of the local stimulus kernel, can be approximated as a convection term by the reaction-diffusion approximation (electronic supplementary material, appendix S3), which prohibits the transmission of the backward pulse (figure 3c,d).

### 3.3. Symptoms in oesophageal motility disorders

Next, we implement various symptoms observed in oesophageal motility disorders.

#### 3.3.1. Lower oesophageal sphincter relaxation failure

In this model, the LES relaxation is induced by CNS signal $S_{\text{CNS}}$. The signal must exceed a threshold to move the toggle switch to another state. Then, SM is relaxed according to the ENS signal propagating from the oral side. Relaxation failure of LES suggests the following parameter changes:

(1) weak signal from CNS to open LES by swallowing (low $i_{\text{CNS}}$, figure 2f);
(2) low LES sensitivity to the negative CNS stimulus (low $T_{\text{ENS}}$ at LES; electronic supplementary material, figure S3a);
(3) abnormally strong smooth muscle contractility (high $B_{\text{SM}}$) at LES region (electronic supplementary material, figure S3f).

#### 3.3.2. Loss of propagation pulse

Several parameter changes in the model can reproduce the disappearance of the peristalsis pulse:

(1) decrease in the sensitivity of the myenteric plexus to the stimulus (high $T_{\text{ENS}}$ at the body; electronic supplementary material, figure S3b);
(2) reduced positive stimulus from CNS (low $s_{\text{CNS}}$, figure 2f);
(3) reduced signal transmissions of interneurons in myenteric plexus (low $s_{\text{IN}}$, figure 2b).

#### 3.3.3. Abnormal pulse speed

Under pathological conditions, transmission speed can be very fast (premature contraction). From mathematical analysis (electronic supplementary material, appendix S3, figure S5), we could obtain the conditions for premature contraction as follows:

(1) increased signal transmissions of interneurons (high $s_{\text{IN}}$; electronic supplementary material, figure S3g);
(2) increased range of projection of interneurons (high $r_{\text{IN}}$; electronic supplementary material, figure S3g);
(3) increased deviation of projection of interneurons to the anal side (high $d_{\text{IN}}$; electronic supplementary material, figure S3g);
(4) increased sensitivity of myenteric neurons to the external stimulus (low $T_{\text{ENS}}$; electronic supplementary material, figure S3b).

#### 3.3.4. High contractility strength

Pathologically strong oesophageal muscle contractions can be implemented as follows:

(1) stronger ENS activity (high $A_{\text{ENS}}$; electronic electronic supplementary material, figure S5c);
(2) stronger ENS motoneuron activity (high $E_{\text{SM}}$; electronic electronic supplementary material, figure S3d);
(3) prolonged smooth muscle contraction (low $D_{\text{SM}}$; electronic electronic supplementary material, figure S3e).

#### 3.3.5. Patterns generated by intraluminal pressure

Our model only includes the effect of CNS, ENS, and SM action, and other physical factors like intrabolus pressure below the peristalsis pulse are not included in this model. Exceptionally, we implement pan-oesophageal pressurization (PEP, simultaneous increase of pressure due to the failure of LES relaxation at food swallowing; electronic supplementary material, figure S3h) *a priori* by directly modifying W(x,t) since it is included in the Chicago classification.

### 3.4. Reproduction of disorders by impaired lower oesophageal sphincter relaxation

Next, we reproduced the pathological oesophageal motility patterns by combining the symptoms described in §3.3.

Oesophageal motility disorders are classified into two groups according to the Chicago classification (electronic supplementary material, figure S1) [3,21,23]. One class is oesophagogastric junction (EGJ) outflow disorders, characterized by the disappearance of a sufficient decrease in IRP. The other group, the disorders of oesophageal peristalsis, is characterized by abnormal peristaltic patterns. At first, we reproduced the disorders characterized by impaired LES relaxation—EGJ outflow obstruction (EGJOO) and achalasia (figure 5).

#### 3.4.1. Oesophagogastric junction outflow obstruction

EGJOO is defined as impaired LES relaxation (figure 5a, red arrow) with relatively intact peristalsis (figure 5a, black arrow) [3,21]. We could reproduce these dynamics by low-amplitude negative stimulus (low $i_{\text{CNS}}$, figure 5b), low excitation threshold at LES (low $T_{\text{ENS}}$ at LES, figure 5c) and high muscle contractility at LES (high $B_{\text{SM}}$, figure 5d). All of these parameter changes resulted in impaired LES relaxation (figure 5b–d, red arrows) with intact peristalsis (figure 5b–d, black arrows).

#### 3.4.2. Type I achalasia

Type I achalasia is defined as impaired LES relaxation and complete failed peristalsis (figure 5e) [3,21]. We could predict from the mathematical analysis that impaired LES relaxation can be reproduced by low-amplitude negative stimulus ($i_{\text{CNS}}$), low excitation threshold ($T_{\text{ENS}}$) at LES and high muscle contractility ($B_{\text{SM}}$) at LES, and the disappearance of the travelling pulse can be implemented by low-amplitude positive stimulus ($s_{\text{CNS}}$), high excitation threshold ($T_{\text{ENS}}$) at body and low interneuron connectivity ($s_{\text{IN}}$). We tried all the combinations of these 3×4 factors (figure 5f–h; electronic supplementary material, figure S8). Interestingly, some combinations are difficult to reproduce the observed pattern (electronic supplementary material, figure S8). One possible explanation is that the effect in the body may influence the LES since the effect is non-local. These characteristics may provide insights into type I achalasia pathogenesis.

#### 3.4.3. Type II achalasia

Type II achalasia shows impaired LES relaxation combined with PEP [3,21]. Due to the oesophageal obstruction at the LES (figure 5i, red arrow), the oesophageal pressure is increased ubiquitously by swallowing food (bolus, figure 5i, red arrowhead). We could reproduce this disorder by implementing PEP (figure 5j–l, electronic supplementary material, figure S59, red arrows) in addition to type I achalasia.

#### 3.4.4. Type III achalasia

Type III achalasia is defined as impaired LES relaxation in combination with premature contraction (figure 5m) [3,21]. Using predictions from mathematical analyses and numerical simulations (electronic supplementary material, figure electronic supplementary material, figure S10), we implemented premature contraction by high interneuron connectivity (high $s_{\text{IN}}$; electronic supplementary material, figure S3g), and LES relaxation failure by low-amplitude negative stimulus (low $i_{\text{CNS}}$, figure 5n), low excitation threshold at LES (low $T_{\text{ENS}}$ at LES; figure 5o and electronic supplementary material, figure S3a) and high muscle contractility at LES (high $B_{\text{SM}}$ at LES; figure 5p and electronic supplementary material, figure S3f).

### 3.5. Reproduction of abnormal oesophageal peristalsis disorders

Next, we reproduce the oesophageal body disorder patterns described in the Chicago classification (figure 6).

#### 3.5.1. Distal oesophageal spasm

Distal oesophageal spasm (DES) is defined by premature contraction (fast wave transmission speed) by the swallowing stimulus (figure 6a, dashed red arrow) with normal relaxation of LES (figure 6a, black arrow) [3,21]. From the mathematical analysis (electronic supplementary material, figure S5), we could predict that the fast pulse speed can be obtained by low excitation threshold (low $T_{\text{ENS}}$ at body), high interneuron connectivity (high $s_{\text{IN}}$), long range of interneuron projection (high $r_{\text{IN}}$) and high deviation of interneuron projection (high $d_{\text{IN}}$). Using this information, we could reproduce the acceleration of pulse speed (figure 6b–e). Interestingly, some parameter sets are unable to generate the expected pattern. For example, high $s_{\text{IN}}$ and high $r_{\text{IN}}$ result in the disappearance of sufficient decrease in IRP (figure 6c,d, red arrows), and the resulting patterns are classified as type III achalasia since the non-local parameter may affect the LES state.

#### 3.5.2. Absent contractility

Absent contractility is defined as complete failed peristalsis with normal LES relaxation by swallowing (figure 6f, black arrow) [3,21]. We could reproduce this state by several methods. Low positive signal (low $s_{\text{CNS}}$), high excitation threshold at body (high $T_{\text{ENS}}$ at body; electronic supplementary material, figure S3b) and low interneuron connectivity (low $s_{\text{IN}}$, figure 2b) result in the disappearance of the pulse as expected (figure 6g–i). We also noticed from clinical consideration that the failure of signal transmission from ENS to SM (low $E_{\text{SM}}$; electronic supplementary material, figure S3d) also results in the disappearance of the pulse (figure 6j).

#### 3.5.3. Jackhammer oesophagus

Jackhammer oesophagus is defined as hypercontractile peristalsis induced by the swallowing stimulus (figure 6k, red arrow) with normal LES relaxation (figure 6k, black arrow) [3,21]. In some cases, the duration of excitation is markedly elongated (figure 6l, red arrow). We reproduced these dynamics by neurogenic and myogenic causes (figure 6m–o). The neurogenic Jackhammer oesophagus was reproduced by increasing the amplitude of excitation $A_{\text{ENS}}$ (figure 6m). Mathematically, amplitude modification is implemented by expanding U nullcline to U direction (figure 3c). This results in the large amplitude and prolonged contraction (the magenta region in figure 6m). Also, the increased contraction amplitude was reproduced by a myogenic cause by increasing the sensitivity of the muscle to the neural stimulus by high neuromuscular signal transmission (high $E_{\text{SM}}$, figure 6n) or low muscular contraction decay (low $D_{\text{SM}}$).

#### 3.5.4. Inefficient oesophageal motility

Inefficient oesophageal motility is defined as over 70% of failed swallows [3,21]. Our model is deterministic, and the same parameter set consistently provides the same result. To implement a stochastic fluctuation, we introduced noise to $T_{\text{ENS}}$, corresponding to the net excitation state controlled by a stochastic autonomic nervous system signal. We considered two situations to implement this effect. One is the basal decrease of parasympathetic nervous system activity $T_{\text{ENS}}$, and the other is interneuron signal transmission strength $s_{\text{IN}}$. We set the initial parameters to induce normal peristalsis even with the addition of stochastic fluctuation (electronic supplementary material, figure S11a). With these parameter changes, among 10 repeated numerical simulations, only two or three initial stimuli were transmitted as a travelling pulse (electronic supplementary material, figure S11b,c), reproducing the observed disorder pattern.

## 4. Discussion

### 4.1. Comparison with previous models

We provided a biologically plausible, analytically manageable model to understand the unique characteristics of oesophageal peristalsis. To our knowledge, this is the first modelling study that deals with the generation of unidirectional pulse transmission of human oesophageal peristalsis. Previously published mechanical models [25–28] define the speed of peristalsis pulse *a priori* as c. Our model can deal with how wave speed is determined. As a result, we could extract various testable hypotheses from the HRM pattern. For example, premature contraction is defined as an abnormally fast transmission of the peristalsis pulse. The previous models cannot deal with the mechanism since pulse propagation speed c is predefined. On the other hand, our model can provide various testable hypotheses as described in §3.

Theoretically, previous mathematical models dealing with non-local kernels did not consider the unidirectional pulse transmission using an asymmetric kernel [29–31].

### 4.2. Bistable behaviour at the lower oesophageal sphincter region

The toggle-switch behaviour (bistability) of oesophageal peristalsis is functionally important but has not been considered. Excitable or oscillatory regimes are frequently used to model neural or muscular behaviour, but the bistable regime is rare [14]. It is known that the internal anal sphincter muscle has a tonic contraction mode [20,32], which suggests the bistable dynamics of the muscle. However, the theoretical model for the tonic mode of the smooth muscle [33] is linear and does not explicitly state bistable dynamics.

### 4.3. Possible experimental verification of the model

The parameters in this model have direct counterparts (interneurons and motoneurons in ENS and smooth muscle), so a pharmacological experiment may be possible if the appropriate experimental system is available. One difficulty is that these three systems share common physiological pathways—serotonergic pathways and cholinergic pathways are both included in interneurons and motoneurons.

There are several obstacles to experimental verifications. First, there are differences between species in the structure of the oesophageal muscle. The human oesophagus consists of the skeletal muscle in the upper one-third and smooth muscle in the lower two-thirds [34]. However, the oesophagus of mice entirely consists of skeletal muscle [34,35] which is cranial origin [36]. Therefore, the experimental results in mice or zebrafish [37] cannot be applied directly to humans. Pharmacological experiments are also difficult for understanding human peristalsis movement for ethical reasons. One possible experiment is the point stimulus of the oesophagus using a balloon [12] while using HRM, which may reveal the unidirectionality of the pulse and the shape of a kernel of the model.

### 4.4. Future directions

There are several possible extensions and applications of the work. Firstly, our model can propose possible causes for various disorders of oesophageal motility, helping to understand the pathogenesis. This approach is helpful since experimental verification is difficult due to differences between species [34,35]. Secondly, our model can be extended to deal with a disorder of the distension phase by modifying $K_{\text{MN}}$ (figures 2c and 4c). Recently, it has been shown that the impaired distension phase underlies the pathogenesis of functional dysphagia [5]. Third, the two-dimensional model is necessary to implement a corkscrew-shaped structure in the Jackhammer oesophagus [38]. In addition, stimulatory and inhibitory neurons can be modelled separately using different kernels, allowing a straightforward description of the effect of a specific pharmacological experiment. The developmental change in oesophageal motility is another intriguing subject. The embryonic gastrointestinal tract is known to show different motility patterns compared with those of adults [39,40].

### 4.5. Limitations of our study

Currently, our model focuses on how peristalsis waves are generated in the ENS, and the generation of intraluminal pressure or the feedback of the mechanical signal to the ENS is not involved, as it is difficult to avoid unintentional excitations. Engineering-type models that focus on the mechanical aspect [25–28] may complementarily address this aspect. In addition, we currently deal with HRM examination conditions, and feedback from food is not yet considered.

## Data Availability

Data are available in [41]. Supplementary material is available online [42].
